# Job Satisfaction among Health-Care Staff in Township Health Centers in Rural China: Results from a Latent Class Analysis

**DOI:** 10.3390/ijerph14101101

**Published:** 2017-09-22

**Authors:** Haipeng Wang, Chengxiang Tang, Shichao Zhao, Qingyue Meng, Xiaoyun Liu

**Affiliations:** 1 School of Health Care Management, Key Lab of Health Economics and Policy, NHFPC (Shandong University), The Centre for Economic Research, Shandong University, Jinan 250100, China; wanghaipeng@sdu.edu.cn; 2 School of Public Administration, Guangzhou University, Guangzhou 510006, China; tang.chengxiang@gmail.com; 3 School of Public Administration, Shandong Normal University, Jinan 250014, China; zhaozhao1988320@163.com; 4 China Centre for Health Development Studies, Peking University, Beijing 100191, China; qmeng@bjmu.edu.cn

**Keywords:** job satisfaction, health-care staff, latent class analysis, township health center, China

## Abstract

*Background*: The lower job satisfaction of health-care staff will lead to more brain drain, worse work performance, and poorer health-care outcomes. The aim of this study was to identify patterns of job satisfaction among health-care staff in rural China, and to investigate the association between the latent clusters and health-care staff’s personal and professional features; *Methods*: We selected 12 items of five-point Likert scale questions to measure job satisfaction. A latent-class analysis was performed to identify subgroups based on the items of job satisfaction; *Results*: Four latent classes of job satisfaction were identified: 8.9% had high job satisfaction, belonging to “satisfied class”; 38.2% had low job satisfaction, named as “unsatisfied class”; 30.5% were categorized into “unsatisfied class with the exception of interpersonal relationships”; 22.4% were identified as “pseudo-satisfied class”, only satisfied with management-oriented items. Low job satisfaction was associated with specialty, training opportunity, and income inequality. *Conclusions*: The minority of health-care staff belong to the “satisfied class”. Three among four subgroups are not satisfied with income, benefit, training, and career development. Targeting policy interventions should be implemented to improve the items of job satisfaction based on the patterns and health-care staff’s features.

## 1. Introduction

Township health centers (THCs) are the hinge of the rural three-tier healthcare network of China, which plays an important role in guaranteeing the health of rural populations and establishing a rural public health system. In China, 1.23 million health-care staff, including clinicians, nurses, and public health practitioners (PHPs), in THCs delivered 1.02 billion outpatient visits in 2013. However, there were only 1.41 health-care staffs per thousand rural populations in 2013, which was far behind that of 9.18 in city [1]. A shortage of health-care staff in rural and underserved areas is a critical issue [2,3]. One main reason for the geographic mal-distribution in health-care staff is the low job satisfaction, which is caused by low income, poor working condition, and limited opportunity of career development in THCs [4,5].

The concept of job satisfaction has a very long tradition. In 1976, Locke defined job satisfaction as a pleasurable or positive emotional state resulting from the appraisal of one’s job or job experience [6], which in later research was redefined as the achievement of one’s job values in the work situation resulting in a pleasurable emotional state [7,8]. Job satisfaction is attracting considerable research in work and organizational psychology. While a variety of definitions of the term job satisfaction have been suggested, this paper will use the definition of “the attitudes towards one’s work and the related emotions, beliefs, and behaviors”, which depends on not only the nature of work, but also the disposition, attitude, and expectation of health-care staff [9,10].

Job satisfaction is seen as a multidimensional variable which is measured based on one’s attitude toward various aspects of the job and job experience. Traditional measurement of job satisfaction is criticized because of the personalistic approach and artificially high proportions of satisfied. The extended model of different forms of job satisfaction is proposed and validated, which is suggested as a distinctive approach to job satisfaction [8]. Six forms of job satisfaction, including progressive, stabilized, resigned satisfaction, and constructive, fixated, resigned dissatisfaction, are derived from the constellation of four constituent variables: comparison of the actual work situation and personal aspirations, global satisfaction, changes in level of aspiration, controllability at work [11]. Therefore, various methods are proved to be useful in evaluating particular aspects of job satisfaction. Latent-class analysis can be used to explore the underlying cognitive and evaluative processes in the formation of different forms of job satisfaction [12].

Job satisfaction is essential for health-care staff’s motivation and performance [13,14]. Job satisfaction can be seen as an indicator of well-being. The anticipation of the development of job satisfaction can be regarded as the estimation of future well-being at the workplace [15]. Better workforce performance is positively associated with higher job satisfaction [16], and value attainment and affective disposition would demonstrate complex interactions with performance and satisfaction [17]. A path model has been tested for the relationship between satisfaction and performance, which highlights an important point that inventions designed to raise job satisfaction may concomitantly increase performance levels [18].

Job satisfaction has a strong inverse relationship with the employee’s turnover intention, and is seen as a major predictor for intention to quit. The correlations between job satisfaction and individual behavior like absenteeism or turnover have been observed in many studies. If job satisfaction is low or the future expectation of job satisfaction is negative, the employees might have the intention to leave the organization [19]. It is confirmed that job satisfaction has only an indirect influence on the intention to quit, whereas organizational commitment has the strongest and most direct impact on it [20,21]. Low levels of job satisfaction will adversely affect employee commitment [22], and sequentially lead to intention to quit and turnover [23,24]. Hence, examining job satisfaction is helpful in developing intervention strategies to reduce turnover intention.

Previous studies have showed that most health-care staff are dissatisfied with their work and working experience in China [5,25,26], and job satisfactions in these studies are investigated based on a variable-centered approach of analysis. However, identifying the dissatisfied health-care staff provides an opportunity for organizations and policy makers to take measures for preventing workforce turnover and improving work performance. To do so, information is required on what dimensions health-care staff in rural China are satisfied or dissatisfied with, as well as their personal and professional characteristics. This requires a person-centered approach of analysis, such as latent class analysis (LCA), which is a statistical method for finding subtypes of related cases from multivariate categorical data [12], and has been used to investigate job satisfaction among doctors in Australia [27] and pharmacists in America [12]. This study is conducted by applying LCA to identify patterns of job satisfaction among health-care staff in rural China, and to investigate the association between the latent clusters and health-care staff’s personal and professional features.

## 2. Materials and Methods

This study utilized data collected from the 2013 China Rural Primary Care Survey that is sponsored by China’s National Health and Family Planning Commission. In order to select a representative sample, the 2013 survey implemented a multistage purposive sampling design. First, three provinces (Shandong, Anhui, Shannxi) were chosen to represent eastern, central, and western China. Further, three counties that can represent different levels of economy and health care development within each province were selected. Finally, five THCs within each county were randomly selected. Overall, forty-five THCs were included in this national survey. In this survey, data collection was based on a voluntary and anonymous principle. The participants were all health-care staff available at the workplace on the day when the investigators visited the THCs. Ethics approval was obtained from the ethics committee of Peking University Health Science Center, Peking University, Beijing, China (Ethical Approval Code: IRB00001052-11083). Verbal informed consent for participation in the survey was obtained from all participants.

The job satisfaction measures in this study basically followed the Warr, Cook, and Wall scale, which includes 16 items covering both intrinsic and extrinsic factors with 15 items, plus overall satisfaction [28]. We excluded three items that were not considered to measure in this survey on job security, staff’s suggestion, and variety in this job. Shi and his colleagues have also examined the factors associated with health-care staff’s job satisfaction by using an urban–rural survey in China [5]. While our version of job satisfaction included two items of relationships with colleagues/fellow and assigned hours of work as extrinsic factors of job satisfaction, instead of receipt of honors/awards and living environment as in Shi’s job satisfaction scale [5,28]. Therefore, the final version of job satisfaction in our study consists of 13 items, including one general satisfaction item (Table 1). Our version of job satisfaction demonstrated good psychometric properties with an overall internal consistency (13 items) of 0.87.

A number of covariates were also collected by the survey: health-care staff’s age, gender, marriage status, perceived importance of training within the THCs (five-point Likert scale), working hours per week, type of health-care staff (PHP, non-PHP), intention to leave the current job (five-point Likert scale), appropriateness of compensation structure within the THCs (five-point Likert scale), permanent job (yes or no), length of working experience, urban residence in childhood (yes or no).

Latent class analysis (LCA) divides health-care staff into different categories by using twelve job satisfaction items [29]. For each individual, the LCA estimates what class the person belongs to. As a result, we also obtained the size of mutually exclusive groups. Mplus 7 was utilized in this study to identify subgroups. First, for the purpose of easy interpretation, the five-point Likert scale of job satisfaction was collapsed into a binary category: dissatisfied (very dissatisfied, dissatisfied, not sure) and satisfied (satisfied and very satisfied). Second, we selected the number of classes by fitting two-, three-, four-, and five-class LC models. The selection criterion considered statistical fit indices such as Bayesian information criterion (BIC), the Vuong–Lo–Mendell–Rubin likelihood ratio test and an entropy measure. The number of classes which yielded lowest BIC was chosen. Third, we specified covariates for use in the adjusted three-step analysis proposed by Vermunt, because this three-step approach can resolve the downward bias problem in a conventional three-step LCA [30]. The following covariates are included: gender, marriage status, perceived importance of training within the THCs, working hours per week, type of providers, income inequality within the THCs, permanent job, length of working experience, urban residence in childhood. Finally, we conducted chi-square analysis to investigate the association between class membership and health-care staff’s intention to leave the current job.

## 3. Results

### 3.1. Descriptive Analysis

Table 2 illustrates the profile of health-care staff in China’s rural THCs. After excluding administrative staff and observations with missing values or incorrect values, the number of valid responses was 603. The majority of health-care staffs in THCs were female (63.85%). The average age of respondents was 36.5 years old, and most of them were living with a partner (88.89%). The proportion of public health providers was 30.85%. Most of them were practicing almost 54 h per week, worked in a permanent job (78.94%), and reported a rural background (73.96%). While the job satisfaction items were recorded in a binary scale, the satisfied percentages for most items were quite low, ranging from 10.95 to 68.49%. Out of all items, only “Fellows” and “Patients” had relatively high satisfied percentages, which were more than 50%. Furthermore, the satisfied percentages of “Income”, “Benefits”, “Training”, and “Career” were less than 20%.

### 3.2. LCA (Latent Class Analysis) Results

In order to select an appropriate number of latent classes, two-, three-, four-, and five-class LC models were fitted to compare the BIC, Vuong–Lo–Mendell–Rubin likelihood ratio test and entropy values. The model fit statistics demonstrated that the four-class model had the lowest log likelihood and BIC, however the entropy value (0.802) remained relatively high. Moreover, the positive predictive values ranging from 0.842 to 0.948 showed the model was suitable in its predictive power (Table 3). Finally, the results revealed that four classes was the optimum number in the latent class model.

**Figure 1:**
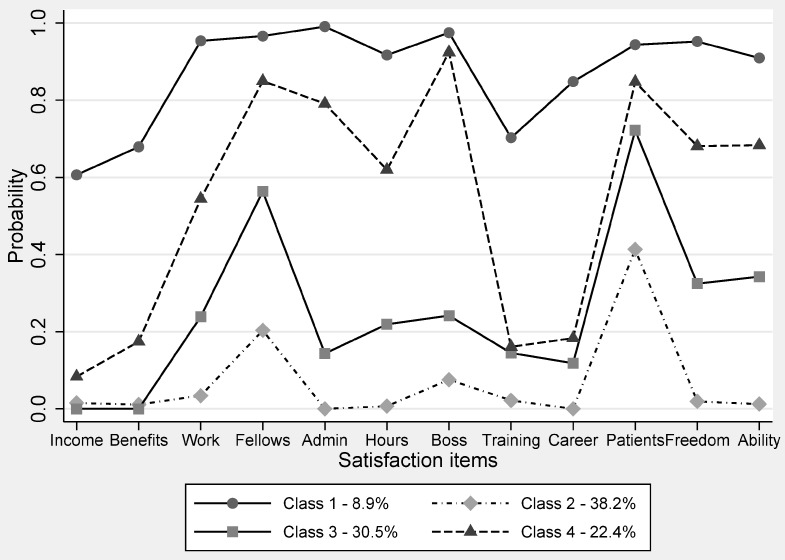
The latent profiles of job satisfaction items.

In this study, four distinct classes of job satisfaction were defined as follows (Figure 1): Class 1 is defined as the “satisfied group”. This class reported the highest probabilities for each job satisfaction item from 0.66 (income) to 1.00 (admin). However, only 8.9 percent of health-care staffs were classified into this class. Class 2 is defined as the “unsatisfied group”. This class was unsatisfied with their job across twelve items, representing 38.2 percent of health-care staff. Class 3 is defined as the “unsatisfied group with the exception of interpersonal relationships”; 30.5 percent of the participants indicated a low satisfaction degree with almost all of the items, but they reported relatively high satisfaction (above 0.5) with only “Relationship with colleagues” and “Recognition from patients”. Class 4 is defined as the “pseudo-satisfied group”; 22.4 percent of health-care staff were categorized into class 4 based on their estimated posterior probability, in which health-care staff were mainly satisfied with management-oriented items. They were generally satisfied with most items of job satisfaction (seven of twelve).

Table 4 presents the associations between covariates and latent class membership. The model was parameterized using reference class 1, so the significantly positive coefficient indicates being married, higher importance of training within the THCs, and being non-PHP staff were significantly associated with the class 1 “satisfied group”. However, health-care staffs who reported stronger income inequality within the THCs were more likely classified into classes 2, 3, and 4. Finally, the chi-square analysis results demonstrated classes 2, 3, and 4 tended to report higher probability of leaving the current job.

## 4. Discussion

This study identified a range of attributes that influence job satisfaction for health-care staff in China’s rural THCs, including extrinsic factors and intrinsic factors. The results show that the majority of health-care staff were dissatisfied with most items of job satisfaction—especially Income, Benefits, Training, and Career development. Moreover, the advantage of this study is that we explored four distinct classes of job satisfaction among health-care staff by employing LCA [12]. Only less than one in ten health-care staff belonged to the “satisfied class”, with high levels of satisfaction in all measured aspects. They can achieve even higher level of job satisfaction by increasing the level of separate aspiration. Nevertheless, sixty percent of doctors belonged to the high satisfaction class in Australia [27,31]. Comparatively, health-care staff had a low level of job satisfaction in rural China.

In contrast, almost four in ten health-care staff belonged to the “unsatisfied class”, with very low levels of satisfaction on most measured aspects. Health-care staff belonging to this group felt dissatisfied with their work situation and experience. Furthermore, more than one in five health-care staffs was categorized into the “pseudo-satisfied group”, who were generally satisfied with most items of job satisfaction (seven of twelve). However, health-care staffs in this group were mainly satisfied with management-oriented items, but dissatisfied with key motivating items such as “income”, “benefits”, “training”, and “career”. It is also indicated that training and career development are closely related to the level of job satisfaction, besides income and benefits. Finally, health-care staffs across four groups were the least satisfied with their income levels and benefits, while they were generally satisfied with patient–physician relationships and those among colleagues. This finding is consistent with previous studies, in which income was also one of least satisfied items [5,32]. It is suggested that most health-care staff in rural China are not satisfied with their income level and career development.

In terms of the association between the class membership and personal and professional features, it was found that higher satisfaction with training opportunities and being non-PHP staff were significantly associated with the “satisfied group”. However, health-care staffs who felt low appropriateness of compensation structure within the THCs were more likely to be categorized into other classes with low level of job satisfaction. One explanation is that health-care staffs in rural China deem the training opportunity as a predictor of career development, and thus health-care staff with more training opportunities will be more satisfied with work. Second, PHPs have heavier workloads and longer working hours after the New Healthcare Reform, so they might report lower job satisfaction if their income level was not increased. Third, after the reform of pay for performance, health-care staffs pay more attention to income disparity within THCs [33], so they would be dissatisfied with the job if they felt low appropriateness of compensation structure within the THCs. Finally, this study also found a positive relationship between low job satisfaction and turnover intension. This confirmed that high-turnover intention is significantly associated with working and security conditions [14]. If health-care staffs have lower job satisfaction, they would have higher intention to leave their working positions.

These findings suggest that Chinese health-care staff in THCs could find their job rewarding in many ways. Thus, there are a range of strategies to address low job satisfaction in order to prevent the undesirable consequences associated with it among health-care staff in rural China. First, programs should be launched to support health-care staff to practice in rural and remote areas. With limited working and living conditions, health-care staff experience low job satisfaction in underserved areas. Second, salary system reform should be made to improve income and benefits for health-care staff practicing in THCs, and the monetary compensation for their work should be based on pay-for-performance [22]. Third, national and local governments should enhance investment in education and training programs that target health-care staff in THCs. Increasing opportunities for career training and professional development is a potential strategy to ameliorate low levels of job satisfaction [34]. These strategies could prevent the undesirable consequences associated with low job satisfaction. The government officials and hospital administrators should pay more attention to influencing factors of job satisfaction and addressing the reasonable needs of rural health-care staff [35]. Both work-related factors and personal features are very important in job satisfaction. Understanding personal features can help to identify sub-groups of health-care staff at particular risk of low satisfaction, while understanding work-related factors can help to identify potential intervention points.

There are a few limitations we must be open to acknowledge in this study. First, the data was self-reported by the participants, which might render some measures less reliable. Second, though we consider this is a representative sample in China because of its rigorous sampling method in collecting data, the representativeness of small sample size may still be challenged and disputed. A more appropriate indication could be that the sample is a representative of the middle economic and health development level in China. Third, this study only conducted correlation analysis, so it would be more interesting to investigate the causality with updated data and more advanced methods in future studies. This study tries to identify patterns of job satisfaction among health-care staff in rural China. Further research can be conducted to investigate the stability of the patterns and possible explanations for a possible non-stability.

## 5. Conclusions

In this study, a latent class model was applied to effectively identify subgroups with heterogeneous job satisfaction among health-care staff in rural China. The minority of health-care staffs belong to the “satisfied class”. Three among four groups are not satisfied with income, benefits, training, and career development. A combination of financial and non-financial incentives should be required to improve job satisfaction among rural health-care staff. Targeting policy-interventions such as financial incentives, supporting professional development, and investment in training programs should be designed and implemented to improve the items of job satisfaction based on the patterns and health-care staff’s features.

## Figures and Tables

**Table 1:** Job satisfaction items in details.

Dimension	Items	Definition
Extrinsic factors	Income	Wage or salary
	Benefits	Benefits and allowances beyond income
	Work	Physical working conditions
	Fellows	Relationship with colleagues and fellow workers
	Administration	Management level within the township health centers (THCs)
	Hours	Assigned hours of work
	Boss	Immediate boss
Intrinsic factors	Training	On-the-job training
	Career	Professional development
	Patients	Recognition for work from patients
	Freedom	Freedom to choose methods of working
	Ability	Opportunity to use abilities

**Table 2:** Characteristicsand job satisfaction of health-care staff in China’s rural THCs. PHP: public health practitioner.

Variables	All (*n* = 603)
Gender	
Male, *n* (%)	218 (36.15)
Female, *n* (%)	385 (63.85)
Age, mean (SD)	36.5 (8.40)
Marriage status	
Single, *n* (%)	67 (11.11)
Living with a partner, *n* (%)	536 (88.89)
Importance of training within the THC, mean (SD)	1.82 (0.91)
Weekly working hours, mean (SD)	53.75 (20.08)
Type of providers	
PHP, *n* (%)	186 (30.85)
Non-PHP, *n* (%)	417 (69.15)
Income inequality within the THC, mean (SD)	2.83 (0.99)
Permanent job	
Yes, *n* (%)	476 (78.94)
No, *n* (%)	127 (21.06)
Length of working experience, mean (SD)	14.66 (8.59)
Urban residence in childhood	
Yes, *n* (%)	157 (26.04)
No, *n* (%)	446 (73.96)
Intention to leave the current job, mean (SD)	2.86 (1.13)
Job satisfaction items in binary	
Income, *n* (%)	66 (10.95)
Benefits, *n* (%)	80 (13.27)
Work, *n* (%)	198 (32.84)
Fellows, *n* (%)	332 (55.06)
Administration, *n* (%)	213 (35.32)
Hours, *n* (%)	198 (32.84)
Boss, *n* (%)	262 (43.45)
Training, *n* (%)	110 (18.24)
Career, *n* (%)	115 (19.07)
Patients, *n* (%)	413 (68.49)
Freedom, *n* (%)	223 (36.98)
Ability, *n* (%)	230 (38.14)

**Table 3:** Average latent class probabilities for most likely latent class membership (Row) by latent class (Column).

	1	2	3	4
1	0.948	0.000	0.000	0.052
2	0.000	0.897	0.102	0.000
3	0.000	0.085	0.842	0.072
4	0.019	0.000	0.072	0.909

**Table 4:** Comparison of health-care staff’s characteristics and attitudes by class (Reference: Class 1).

Characteristics/Attitudes	Class 2		Class 3		Class 4	
	Coefficients	S.E.	Coefficients	S.E.	Coefficients	S.E.
Gender	−0.124	0.468	−0.337	0.471	−0.705	0.469
Marriage status	1.506 **	0.690	1.408 **	0.673	1.405 **	0.708
Urban background	0.237	0.553	0.261	0.557	0.476	0.566
Weekly working hours	0.005	0.019	0.016	0.017	0.002	0.017
Specialty	0.795 ***	0.231	0.568 **	0.222	0.414 *	0.225
Permanent job	−0.240	0.752	−0.727	0.731	−1.004	0.736
Length of working experience	−0.006	0.157	−0.068	0.156	−0.098	0.154
Appropriateness of compensation structure within the THC	−2.740 ***	0.387	−2.234 ***	0.401	−1.403 ***	0.403
Higher importance of training within the THC	1.169 ***	0.237	0.909 ***	0.235	0.566 ***	0.223

* *p* < 0.1; ** *p* < 0.05; *** *p* < 0.01.
